# Emerging electronic deodorization technologies for human odor management

**DOI:** 10.4103/mgr.MEDGASRES-D-25-00127

**Published:** 2026-01-06

**Authors:** Solpa Lee, Pratiksha Diwe, Tae Ho Lim, Yongwoo Jang

**Affiliations:** Department of Medical and Digital Engineering, College of Engineering, Hanyang University, Seoul, Republic of Korea; Department of Emergency Medicine, Hanyang University Hospital, Seoul, Republic of Korea; Department of Pharmacology, College of Medicine, Hanyang University, Seoul, Republic of Korea

Deodorization refers to the removal or neutralization of unpleasant odors, including those originating from the human body. With the increasing emphasis on personal hygiene and social interaction in modern society, the management of human body odor has become an important issue in both the healthcare and consumer product sectors. The metabolic activity of the skin-resident microbiota is the main cause of human body odor. Although sweat itself is odorless, it contains non-volatile precursors that are metabolized by skin microbiota, including *Propionibacterium*, *Micrococcus*, *Staphylococcus*, and *Corynebacterium* species, into volatile metabolites responsible for body odor.^1^ In particular, axillary odor is strongly influenced by sulfur-containing compounds such as 3-methyl-3-sulfanylhexan-1-ol (3M3SH) and branched-chain fatty acids such as 3-hydroxy-3-methylhexanoic acid (HMHA).^2^

Demand for efficient and long-lasting deodorization techniques is rising as a result of the psychological pain and social stigma attached to offensive body odors. Conventional approaches primarily attempt to prevent perspiration, mask odors, physically absorb odors, or inhibit bacteria. However, these methods generally offer only short-term efficacy and can adversely affect the natural skin microbiome. Furthermore, their non-reusable nature and short application intervals restrict both practicality and environmental sustainability. These shortcomings highlight the need for next-generation deodorization solutions, especially those integrating biotechnology and electronics, such as electronic deodorization technologies.

**Figure 1:**
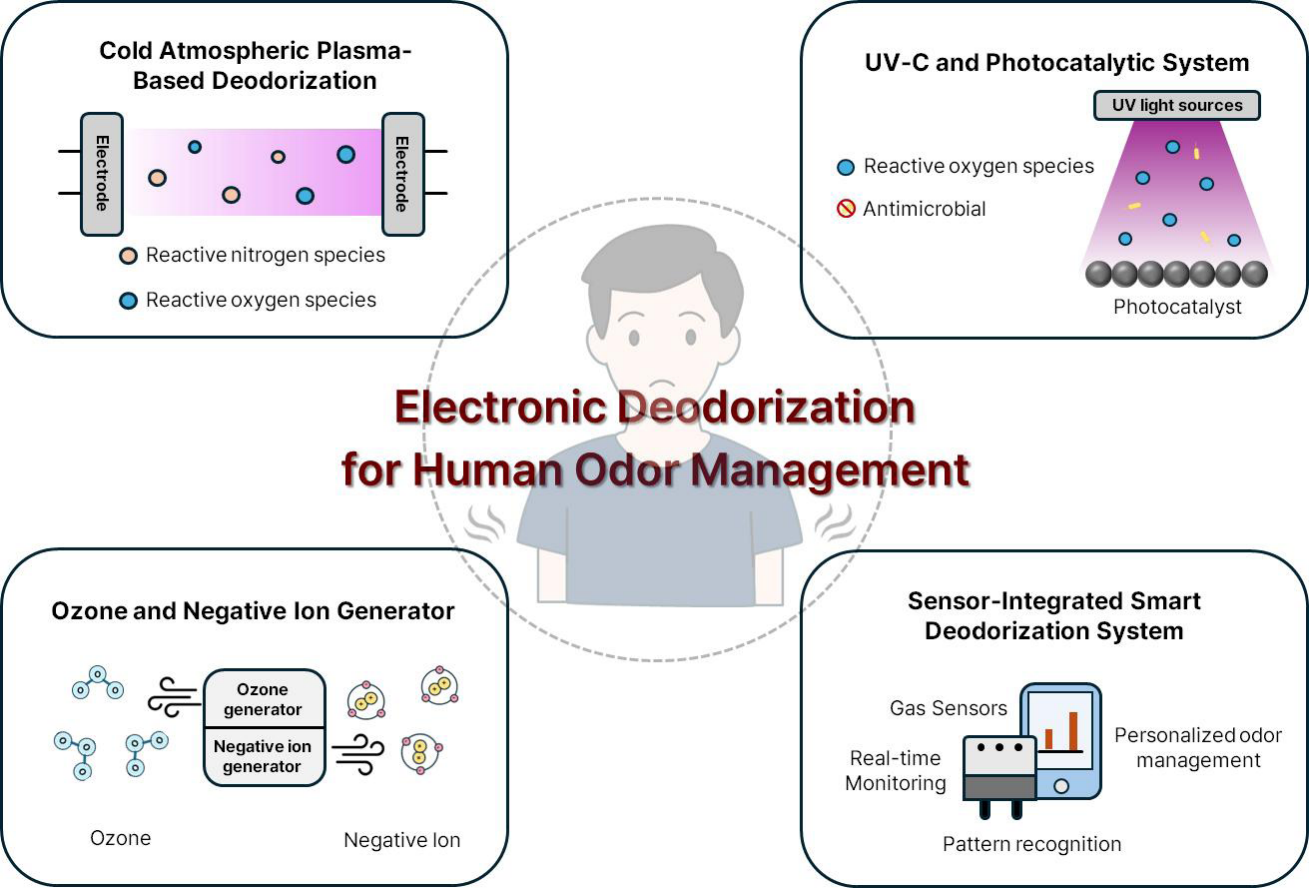
Emerging electronic deodorization technologies for human odor elimination. (1) Cold atmospheric plasma-based deodorization through RNS and ROS generation; (2) UV-C and photocatalytic systems that generate reactive oxygen species for oxidative degradation of volatile organic compounds and their antimicrobial function; (3) ozone and negative ion generators for oxidizing odorous molecules and airborne particulate reduction; (4) sensor-integrated smart deodorization systems to monitor deodorization efficacy. Created with Microsoft PowerPoint. RNS: Reactive nitrogen species; ROS: reactive oxygen species; UV: ultraviolet; UV-C: ultraviolet-C.

Electronic deodorization refers to the use of electrically driven systems to remove or neutralize odorous compounds through physical, chemical, or biological processes (**Figure 1**). These technologies provide targeted, reusable, and environmentally sustainable solutions for odor control. Cold plasma technologies, ultraviolet-C (UV-C)-driven photocatalytic systems, and systems generating ozone or negative ions were most recently introduced, offering different mechanisms for degradation or neutralization of odorous compounds (**Table 1**).

**Table 1:** Comparison of electronic deodorization technology

Electronic deodorization technology	Deodorization agent	Antimicrobial effect	Safety consideration	Application
Cold atmospheric plasma-based deodorization	Reactive species	o	Generally safe for skin	Direct skin application, *in situ* treatment
Ultraviolet-C and photocatalytic system	Ultraviolet C photons, photocatalysts	o	Harmful to skin/eyes, indirect use recommended	Clothes, fabrics, and accessories sterilization, enclosed environment
Ozone and negative air ions	Ozone molecules and negative air ions	o	Safety concerns with prolonged exposure or high doses	Wearables, compact device, enclosed environment
Sensor-integrated smart deodorization system		x	No chemical risk	Real-time monitoring, personalized odor management

"o" denotes the presence of antimicrobial activity, whereas "x" indicates its absence.

**Cold atmospheric plasma (CAP)-based deodorization systems:** CAP is a quasi-neutral ionized gas generated through the ionization of ambient air. As a type of non-thermal plasma, CAP is composed of energetically excited particles that are not in thermal equilibrium, which allows it to function under atmospheric pressure.^3^ CAP can be created from compact and portable devices, therefore reduces operating costs and can be utilized in situ. Additionally, its benign interactions with biological tissues allow it to be directly applicate to cells and skin surfaces, supporting its use in a broad range of medical and therapeutic contexts.^4^

During a plasma discharge, there are abundant oxygen and nitrogen molecules found in the air that become ionized and cause a transient increase in the reactive oxygen and nitrogen species (reactive oxygen species [ROS] and reactive nitrogen species [RNS], respectively).^5^ In addition to these radicals, plasma discharge generates a complex mixture of biologically active components including charged particles, neutral species, ozone, and ultraviolet (UV) radiation. Reactive species and charged particles are the key components of CAP-mediated sterilization and disinfection.^6^ The antibacterial effect of CAP is primarily mediated by ROS and RNS as reactive radicals, which cause oxidative stress in microbial cells. In addition to its antimicrobial properties of CAP, the existence of highly reactive chemical species is essential in the degradation of volatile organic compounds (VOCs) that are major contributors to malodors. Reactive species, such as ozone that is generated from the oxygen in the air during plasma discharge, initiate oxidation reactions resulting in the breakdown of VOCs into smaller, and non-odorous byproducts such as carbon dioxide and water.

Notably, a recent study showed that CAP treatment can significantly lower the amounts of certain compounds associated with axillary odor, especially those with butane, hydroxyl, and sulfur groups.^7^ The study also confirmed the deactivation of *Staphylococcus hominis* and *Corynebacterium xerosis*, which are known for major contributors of body odor. These results provide preliminary evidence that both the CAP-mediated chemical degradation of VOCs and its antimicrobial effects play pivotal roles in human odor reduction. For axillary odors, which arise from the microbial decomposition of sweat components, the ability of CAP to simultaneously deactivate odor-causing bacteria and degrade malodorous compounds highlights its value as a targeted and comprehensive deodorization approach. This dual mechanism underscores the importance of CAP treatment as a non-chemical, residue-free strategy with strong potential for practical applications in deodorant technologies.

**UV-C and photocatalytic systems:** UV-C is a type of ultraviolet radiation within the wavelengths between 100–280 nm and is widely known for its germicidal and oxidative abilities. In the context of deodorization, UV-C irradiation breaks down malodorous compounds and odor-causing microorganisms through a process called photo-oxidation. High-energy UV-C photons generate ROS, such as hydroxyl radicals and superoxide anions.^8^ These ROS oxidize VOCs and deactivate odor-causing microorganisms, ultimately turning them into harmless byproducts such as water and carbon dioxide. Additionally, UV-C exhibits strong antimicrobial activity by inducing DNA damage to microbes, leading to cell death. Photocatalytic systems are often employed with UV-C light, which serves as an energy source to activate photocatalysts. Upon UV irradiation, photocatalysts absorb photons, leading to the excitation of electrons and the formation of electron–hole pairs, thereby generating ROS that participate in the oxidative degradation of odorants. A typical UV-C-based photocatalytic deodorization system comprises three main components: a photocatalyst, a light source, and a photocatalytic reactor. The photocatalyst serves as the reactive surface for the degradation of odorous compounds and is typically composed of titanium dioxide (TiO_2_), although other materials such as metal oxides and metal sulfides have also been investigated to improve effectiveness under various light conditions.^9^ The light source is usually a UV-C light-emitting diode that provides the photon energy required to activate the photocatalyst. While UV-C and photocatalytic systems exhibit strong potential for eliminating human odor through oxidative and antimicrobial mechanisms, their direct application to the human body is limited by safety concerns such as the risk of skin and eye irritation upon UV-C exposure. Nevertheless, these technologies can be effectively utilized for deodorizing odor-retaining items. For example, photocatalytic textiles created by embedding photocatalytic materials within fibers or applying them as surface coatings, provide self-cleaning and deodorizing capabilities. In addition, their integration into enclosed environments such as personal lockers, footwear compartments, and small living spaces represents a promising strategy for safely managing human-derived odors.

**Ozone and negative ion generators:** Ozone is a triatomic molecule composed of three oxygen atoms, with the third atom weakly bound, making the molecule highly unstable and reactive. Due to its instability, ozone tends to be generated in situ and will spontaneously dissociate within a few minutes. When applied at low concentrations, such a transient nature renders ozone relatively safe for use in human odor control applications. Ozone can be generated by ultraviolet radiation or, more often, through a corona discharge system, where high-voltage electricity is applied across electrodes separated by an insulating material. This creates an electric corona that dissociates molecular oxygen (O_2_) into atomic oxygen (O), which then recombines to form ozone (O_3_). In the context of deodorization, ozone reacts with VOCs and odor-causing microbes located on the skin surface. The reactive third oxygen atom begins an oxidation reaction that irreversibly deconstructs the malodorous compounds into benign byproducts including oxygen, water, and trace amounts of nitrogen dioxide, thereby permanently eliminating the cause of the odor.^10^ In addition to its deodorizing function, ozone acts as an antimicrobial agent by oxidizing microbial cell membranes and intracellular components, resulting in structural damage and cell death. Ozone-based deodorization is a cost-effective and space-efficient solution owing to the simplicity of ozone-generation systems and the absence of consumable chemical agents. These characteristics make ozone particularly suitable for integration into compact, portable devices designed for personal use such as deodorizing modules embedded in wearables, small home appliances, and travel accessories.

Negative air ions (NAIs) are small, negatively charged particles found in the atmosphere, mostly in the form of superoxide anions (O_2_^−^). NAIs are typically generated when gas-phase molecules or atoms absorb high-energy inputs such as electrical discharges or cosmic radiation, leading to the ejection of electrons and the attachment of free electrons to neutral molecules.^11^ NAI generators usually work using a corona discharge to release electrons into the air. These free electrons subsequently interact with oxygen and water vapor, forming reactive ions such as superoxide and hydroxyl species. These ions are potent oxidizers capable of decomposing VOCs, airborne microbes, and inhalable particulate matter. Through these oxidative reactions, NAIs can help remove odor-causing substances from the environment. The direct application of NAIs to the human body may provide deodorization benefits by reducing odorant molecules and suppressing microbial growth on the skin. However, prolonged or excessive exposure to high concentrations of NAIs may pose potential health risks, highlighting the importance of controlling emissions for safe and effective use in the management of human odors.

**Sensor-integrated smart deodorization systems:** Smart deodorization systems with gas sensors provide real-time monitoring and automated process control, thereby providing effective and responsive odor management solutions. Although gas sensors with high selectivity towards specific human odor-causing compounds are currently unavailable, the electronic nose technology, which can recognize complex patterns of VOCs provides a promising alternative for assessing deodorization performance.^12^ These systems typically incorporate various types of sensors such as metal oxide semiconductor sensors, electrochemical sensors, and optical sensors. They are combined with pattern recognition algorithms, including machine learning approaches, to detect subtle changes in VOC profiles before and after deodorization. However, achieving high selectivity for specific human odor compounds in real-time remains a major challenge due to the complexity and variability of odor mixture. To overcome this limitation, advanced data-driven approaches can be employed to enhance specificity and robustness. By adding such a system to deodorization devices, users can optimize operating parameters, such as the exposure time and intensity, and quantitatively ensure deodorization efficacy. Furthermore, real-time feedback enables personalized odor management that is tailored to individual needs and environmental conditions. Thus, such smart systems represent a practical and user-friendly solution for the continuous evaluation and enhancement of human odor control technologies.

In conclusion, electronic deodorization technologies represent a next-generation strategy for human odor management that provides non-chemical, efficient, and intelligent alternatives to conventional methods. Such technologies utilize a range of physical, chemical, and biological mechanisms such as UV-C irradiation, photocatalysis, ozone generation, cold plasma treatment, and negative air ionization. As a result, they enable targeted and reusable deodorization with minimal environmental impact and reduce the disruption of the natural skin microbiome. However, safety considerations must be carefully addressed for each technology. For instance, ozone and NAI may pose health risks depending on exposure duration and concentration levels. UV-C irradiation, while effective, can be harmful with direct application to the skin and is thus better suited for use in accessories or indirect treatment strategies. Therefore, optimizing operating parameters and identifying appropriate application scenarios are crucial to ensure both efficacy and user safety in real-world use. With the ongoing progress in enhancing sensor performance, safety optimization, and system miniaturization, electronic deodorization platforms are expected to play an increasingly important role in personal care, clinical hygiene, and daily applications across residential, occupational, and wearable environments.

*This work was supported by the National Research Foundation of Korea (NRF) grant funded by the Ministry of Science and ICT (MSIT) of the Korean government (RS-2023-00302751 and RS-2025-00560524) and project for Collabo R&D between Industry, University, and Research Institute funded by Korea Ministry of SMEs and Startups in 2025 (RS-2025-02315436) (to YJ)*
